# Decarbonizing the Spanish Health System: A Qualitative Study on the Implementation of Environmental Regulations and Management Strategies in Health Institutions

**DOI:** 10.3390/healthcare14060753

**Published:** 2026-03-17

**Authors:** Laura Montes-Piña, Bárbara Badanta, Rocío de Diego-Cordero

**Affiliations:** Research Group PAIDI CTS-1149, Comprehensive and Sustainable Health, Bio-Psycho-Social, Cultural and Spiritual Approach to Human Development (DesH-Global), Faculty of Nursing, Physiotherapy and Podiatry, Universidad de Sevilla, 41009 Seville, Spain; rdediego2@us.es

**Keywords:** climate change mitigation, environmental management, health institutions, health policy, sustainable healthcare

## Abstract

**Background/Objectives**: The healthcare sector, despite its mission to protect health, is a major consumer of resources and emitter of greenhouse gases, giving rise to an ethical and governance paradox: how to reconcile the duty of care with the environmental impact of its activities. In the Spanish healthcare system, which is highly decentralized and regulated at multiple levels, this tension shapes the implementation of environmental policies. This study analyzes the governance and implementation of environmental regulations in Spanish healthcare institutions and the associated experiences. **Methods**: A qualitative, exploratory, descriptive study was conducted using effective meetings and semi-structured interviews with 20 participants, working in healthcare provision and environmental management within health institutions, across different regions of Spain, between September 2024 and November 2025. In addition, a documentary analysis of relevant regulations was undertaken. **Results**: The results indicate that Spanish healthcare institutions improve their environmental performance through the implementation of standards such as ISO or EMAS, although their adoption varies according to each institution’s level of development in environmental management. In addition, differences were observed in the environmental dynamics of healthcare institutions, linked to the decentralization of the Spanish healthcare system, as well as administrative barriers to accessing funding and gender disparities in environmental leadership. **Conclusions**: The standardization of environmental regulations and measures across the country, along with strengthening organizational capacity, could strengthen progress toward more sustainable healthcare.

## 1. Introduction

The health-climate nexus represents a major challenge for contemporary societies. Environmental factors go beyond individual health, impacting global health, that is influenced by the social and cultural context, which requires collaboration with different disciplines, such as anthropology and engineering, in order to reduce health inequalities that extend beyond national borders [1].

According to the World Health Organization (WHO) and the European Environment Agency, climate change threatens population health, increasing the risk of infectious and non-communicable diseases, such as cardiovascular, respiratory, neurological diseases and premature birth. In addition, poor air quality can cause up to 200,000 deaths per year in Europe [2,3].

Furthermore, due to climate change, extreme weather events have increased over the last four decades, with heavy rainfall and extreme heat becoming especially relevant. In 2024, almost 2 million people were affected by floods in Europe and, in Spain, more than 200 people died as a result in the same year [3]. Similarly, high temperatures have led to an increase in heat-related mortality of up to 71.5% in 2025, compared with the previous year, according to the Daily Mortality Monitoring System (MoMo) [4].

This relationship between environment and health is especially important given that human activity is recognized as a major cause of environmental deterioration [3], particularly activities carried out in the health sector. Health institutions consume a large amount of resources such as water, energy, metals and chemical compounds, and generate a large amount of waste and greenhouse gas (GHG) emissions, such as CO_2_, nitrous oxide or methane, reaching up to millions of tons of GHGs and contributing to reduced quality of life through disability, as observed in developed countries [5,6,7]. In this context, if the health sector were a country, it would be the fifth-most polluting country on the planet [8,9]. These data highlight the ethical dilemma inherent in the health–healthcare sector nexus, the latter bearing the moral obligation to do no harm [10,11]. There is therefore a need to transform strategies within the healthcare sector in order to protect human health [12]. The development of policies and regulatory strategies are identified as a central pillar to achieve a more environmentally responsible healthcare system [13,14,15].

In this regard, the European Green Deal, adopted in 2019, serves as a regulatory framework to achieve climate neutrality, including the health sector in efforts to reduce environmental risks and promote the protection of population health [16]. Spain, as a member state of the European Union (EU), had already committed to reducing its environmental impact under the Kyoto Protocol, which establishes that each signatory country “must adopt national policies and measures to reduce GHG emissions” by distributing the effort among the most environmentally significant sectors [17]. Given that the health sector is largely responsible for environmental degradation, Law 7/2021, of 20 May, on climate change and energy transition, was adopted in Spain, which obliges health institutions to adopt environmental responsibilities by developing sustainable management strategies [18].

Although progress has been made in the development of environmental policies within the healthcare sector, few studies have explored how health authorities interpret these mandates and the outcomes achieved through their implementation in a highly decentralized system [12,15,19]. Therefore, this study aims to investigate how Spanish health institutions implement environmental strategies and regulations, as well as the experiences associated with this process.

## 2. Materials and Methods

This study is part of a broader research project entitled “Development of an ethical and gender-sensitive model for the implementation of sustainability measures in healthcare centres and institutions” (SUSTAinsHealth), funded by the VII Research and Transfer Plan (PPIT) of the University of Seville, carried out in two phases between 2024 and 2025.

### 2.1. Design

A qualitative, exploratory, descriptive and narrative study was conducted using a phenomenological approach. This approach aims to describe the meaning of an experience by identifying essential subthemes and overarching themes [20].

### 2.2. Setting, Sample and Eligibility Criteria

Participants belonging to the field of environmental management in the health sector and health professionals working in clinical care and/or management roles within health institutions, both public and private, were included. While data saturation may be achieved with a smaller number of participants [21], in this study, it was necessary to include 21 participants who represented different peninsular and island Autonomous Communities (AACC) of the country: Andalucía, Cataluña, Comunidad Valenciana, Navarra, Madrid, Región de Murcia and Islas Canarias.

Spain is structured into territories that form 17 AACC, distributed across the north, south, east, west and centre of the peninsula, as well as islands belonging to Spanish territory, and two autonomous cities. Each of the Autonomous Communities and Autonomous Cities is responsible for the health competencies of their region, which characterizes the National Health Service (NHS) as highly decentralized. Together with the public NHS, the private health system coexists, thus forming a Beveridge-type health model.

### 2.3. Procedures

Participants were recruited from various geographical areas, belonging to the north, south, east, southeast, centre of the country and island territories. The data were collected by conducting effective meetings with health professionals as a first approach to environmental sustainability in the health sector. The effective meetings consisted of formal meetings that allow the format and means of communication to be adapted, depending on the needs of the participants, improving and optimizing the encounter [22,23]. In this way, the rigidity between the participant and the researcher was reduced, creating a more relaxed and open dialogue, keeping the focus on the topics relevant to the study.

After the completion of the effective meetings, a second phase was launched involving health professionals and staff dedicated to the environmental management of health institutions, through semi-structured interviews, to enable a more in-depth exploration of data related to environmental management.

The conduct of effective meetings and semi-structured interviews were conducted by the authors, whose occupations at the time of the study were researchers at the University of Seville and a registered nurse. There was no prior relationship with the participants or any direct affiliation with the environmental management areas. The data collection was carried out between September 2024 and November 2025, and purposive and snowball sampling strategies were used to select the participants [24]. Snowball sampling was initiated through a search of the official websites of hospitals and health services, in order to identify environmental management units or professionals with experience in the field, who subsequently facilitated contact with other colleagues. For the execution of both, prior contacts (telephone and/or video calls) and written contacts were made, by sending a letter of introduction via e-mail providing project information about SUSTAinsHealth, and the recipients were invited to participate, as well as being sent the programme (date, time, estimated time and means of communication). For the development of the effective meetings open, face-to-face discussions were conducted. Once each meeting was concluded, an executive summary was prepared with the main findings within 24 h, in line with the characteristics of the effective meetings [23].

To carry out the interviews, a script developed and validated by experts was used as an instrument. In this case, the meetings were held either in person or online, depending on participants’ requirements.

To enrich the data analysis, the effective meetings and interviews were combined with the analysis of normative documentation (grey literature). This was provided in physical and verbal form by the participants, who, where necessary, indicated how and where it could be accessed. Once obtained, it was classified and organized according to the topics addressed, following a consecutive and flexible process for its analysis.

### 2.4. Instrument

The authors developed a script for conducting the semi-structured interviews (Table 1), based on the open conversations carried out in the effective meetings and on a previous study on the role of health institutions in the development and implementation of sustainable strategies [6]. Subsequently, the script was evaluated by five experts in clinical management and/or the environment (Table 2), using the Delphi technique in two rounds [25].

### 2.5. Data Analysis

A phenomenological framework based on Amadeo Giorgi’s theoretical model [26] guided the analysis of the data. Giorgi’s approach aims to uncover and articulate the meanings that individuals attribute to the phenomenon under study. In addition, thematic analysis of the data followed the approach described by Braun et al. [27]: (a) familiarization with the data; (b) generation of categories; (c) search, review, and definition of themes; and (d) production of the final report.

Transcription, literal reading, and manual thematic categorisation were performed. The authors repeatedly read and re-read participants’ statements to gain familiarity with the dataset. Data analysis continued by organizing descriptive labels, focusing on emerging concepts and similarities/differences in participants’ statements to obtain the following themes: (1) Regulatory framework; (2) Governance; (3) Environmental analysis and financing; (4) Interinstitutional collaboration for environmental management.

The analysis of grey literature (defined as regulations referred to by participants and not accessible through scientific sources) was carried out in a structured and systematized manner across six phases (collection, classification, organization, analysis, interpretation and presentation) [28], adapted to the specific characteristics of this study.

### 2.6. Trustworthiness

This research followed the Consolidated Criteria for Reporting Qualitative Studies (COREQ) [29]. Methodological triangulation was used, including the use of effective meetings, semi-structured interviews and documentary analysis, as well as data triangulation through the inclusion of participants with different professional profiles (see Supplementary Material, Table S1).

### 2.7. Ethical Considerations

The study was approved by the Andalusian Research Ethics Committee, Spain (Code: 2025-1228). All participants received information about the study, both orally and in writing. All participants included in the study signed an informed consent form.

## 3. Results

The results obtained derived from the completion of five effective meetings and 16 semi-structured interviews, including a total of 21 participants and the analysis of 10 documents relating to regulations and management strategies. Regarding the characteristics of the participants (Table 3), the sample included nine women and 12 men. Participants were healthcare professionals (*n* = 4) and environmental managers, including meso-level management (*n* = 15) and macro-level management (*n* = 2) within the healthcare sector. The institutions of origin were a private healthcare centre (*n* = 1), Occupational Risk Prevention Centers (ORPC) (*n* = 2), hospitals of the National Health System (NHS) (*n* = 14) and central health services of different Autonomous Communities (*n* = 2).

**Figure 1:**
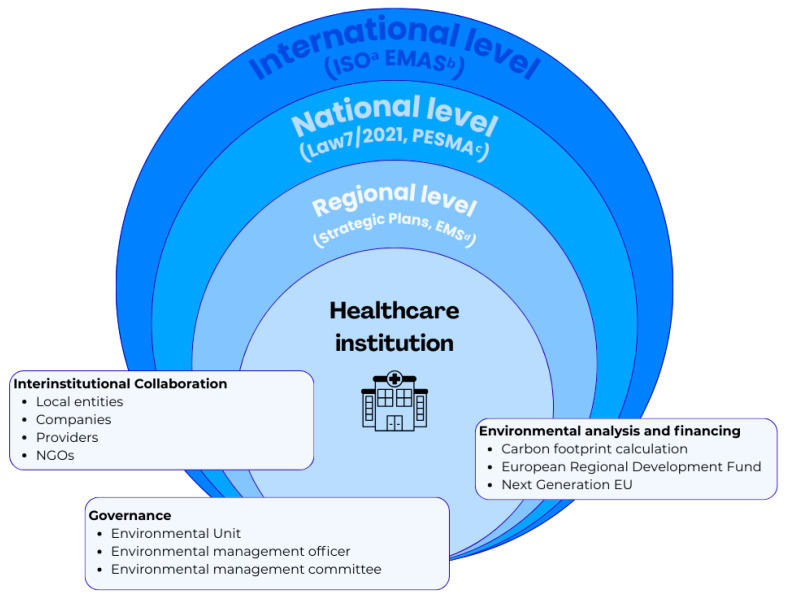
Conceptual model of environmental regulation in Spanish healthcare. Prepared by the authors based on the information analyzed. ^a^ International Organization for Standardization; ^b^ Eco-Management and Audit Scheme; ^c^ Strategic Plan for Health and the Environment; ^d^ Environmental Management System.

The results obtained are shown in Figure 1, which represents the conceptual model of environmental regulation in Spanish healthcare.

### 3.1. Regulatory Framework

Different international, state and regional guidelines govern the management of environmental sustainability in the healthcare sector.

Internationally, most health centres (*n* = 12) are certified under the standards issued by the International Organization for Standardization (ISO), specifically ISO 14001:2015 [30] for environmental management, and up to 10 participants reported that their health centres also apply ISO 50001:2018 [31] for energy management. While both standards offer a systematic approach to organizational performance, following the PHVA cycle of continuous improvement (Plan, Do, Verify, Act), ISO 14001:2015 provides guidelines for institutions on the implementation of an Environmental Management System (EMS), with the aim of reducing their environmental impact [30]. Similarly, ISO 50001:2018 establishes guidelines for the development of an Energy Management System (EnMS), with the aim of improving energy efficiency [31].

The implementation of both standards is voluntary, but institutions that adopt them must commit to developing, maintaining and evaluating an EMS and an EnMS, led by organizational management, and subject to internal and external audits at regular, though not strictly defined, intervals.

An advantage of the ISO 14001:2015 standard is that it allows progress towards other environmental models such as the Eco-Management and Audit Scheme (EMAS). This regulation, also voluntary, incorporates the characteristics of ISO 14001 together with additional requirements that place greater emphasis on transparency, communication and external verification, elevating healthcare towards a model of care that is less harmful to the environment and health “EMAS is very demanding in terms of legal requirements” (I-7, environmental management technician). “I would have liked to move from ISO 14001 to EMAS; it is a much more comprehensive certification and comes closer to excellence (…) but with the resources we have, we can only go so far” (I-13, Environmental Management Officer). Considering its requirements, only two hospitals in this study have opted for the EMAS regulation (Virgen de las Nieves University Hospital and Virgen Macarena University Hospital, both located in southern Spain).

This regulation requires an annual declaration of environmental performance, its publication, and official registration through national entities, such as the General Directorate of Environmental Quality and Climate Change. Both the environmental statement and the external audit of the institution must be carried out by an environmental verifier (individual or organization), who is accredited by national entities or by bodies designated by any EU Member State for this purpose [32] (Table 4).

At the national level, Law 7/2021 of 20 May on climate change and energy transition aims to reduce GHG emissions, improve energy efficiency and promote the adoption of renewable energies through the legislation of the activities of organizations and institutions in Spain. In this way, it commits Spanish organizations and institutions to incorporating environmental responsibilities into their management practices.

Law 33/2011 of 4 October on General Public Health establishes the framework to protect and promote population health from a preventive and collective perspective, reinforcing the role of public health, including the healthcare system, in addressing environmental risks. Within this framework, the Strategic Plan for Health and the Environment (PESMA) was developed, whose objective is to integrate public health and current environmental policies in order to reduce health risks associated with environmental factors. The PESMA focuses its action on fulfilling the European Union’s commitments, such as the European Green Deal and the 2030 Agenda, through research, information and health impact assessment, the promotion of healthy and sustainable environments and lifestyles, and the strengthening of coordination between the Autonomous Communities [33].

Although national regulations apply across the entire territory, each AACC develops its environmental management strategic plans differently, addressing multiple dimensions ranging from governance and sustainable organization; sustainable infrastructure, energy and equipment; clinical processes and sustainable patient care; mobility, purchasing and responsible waste; to people, communication and training. Nevertheless, they all share the common objective of achieving a more decarbonised healthcare system, as demonstrated by plans developed in regions in the north, south, northeast and southeast of Spain.

In the healthcare institution itself, environmental and energy management are incorporated into an EMS developed by the regional healthcare service, within which policies and strategies are defined and environmental guidelines are implemented operationally in health centres. According to the results obtained, the level of EMS implementation may be influenced by institutional sustainability maturity, understood as the time and accumulated experience in this area. In this regard, highly implemented EMSs are found in public healthcare centres in southern Spain, where experience in environmental management has been sustained for more than ten years [34]. However, each of them is applied differently, depending on the specific characteristics of the healthcare institution concerned: “…we have our own internal procedures that determine how things are done in this hospital, which may differ from those in other hospitals’ (I-6, Deputy Director of Economic, Administrative and General Services)”.

### 3.2. Governance

To implement strategic plans in hospitals, there is a largely standardized hierarchical organizational structure. The structure is led by the management directorate, which delegates functions to the sub-directorates. Within this structure, there are usually the economic and general services departments, responsible for environmental management, although participants perceive this responsibility as largely shared across the organization. “Environmental management is not confined to a single department (…) it is fully cross-cutting” (I-11, Deputy Director of Industrial Processes and Services).

In the healthcare facilities analyzed, responsibility for environmental management was more frequently held by men. Although participants did not identify gender as a selection criterion, this predominance of men may be related to the type of functions associated with the position, which involve management and decision-making. Nonetheless, women were also found in these roles, indicating that this is not a formal rule but rather a trend observed in practice. Furthermore, the professional profiles observed in environmental management positions were diverse, with no uniformity across institutions. Despite this diversity, participants agreed on the importance of having specific training in environmental management, which is not always formally required for the position. This situation reflects a certain lack of definition regarding the required professional profile: “The person responsible for environmental management in a hospital must have some knowledge of environmental management... The requirement is to have a university degree” (I-7, environmental management technician).

The responsibility for this task entails implementing, maintaining and evaluating the EMS of the associated centres, as well as providing staff training. In the southern region of the country, for example, training is a key element for professional accreditation by the Andalusian Agency for Healthcare Quality (ACSA). The ACSA provides an accreditation system for healthcare professionals in which sustainable practices in their healthcare activity are assessed, among others, in order to obtain a certain level of competence and advance in their professional careers [35]. “(…) the professional accreditation standards established by the Andalusian Agency for Healthcare Quality include reflective reports related to environmental management” (I-4, Head of the Occupational Risk Prevention Service).

Some institutions in the south of the country also have an energy manager responsible for monitoring and continuous improvement of the EnMS, as well as an environmental management technician. However, in areas of northern and eastern Spain, there are institutions with single-person environmental management departments. The addition of other profiles allows for the establishment of an environmental management committee, in which objectives, areas for improvement, and non-conformities can be discussed.

This committee usually varies its meeting frequency depending on the needs identified in each institution and can be made up of non-clinical departments (general services management, sub-directorate of industrial processes and services, hotel services, maintenance, quality department) and clinical departments (medical and/or nursing management). The latter are considered essential for incorporating the clinical perspective into the committee and for facilitating the transfer of information to the rest of the staff in clinical departments.

Even in some institutions in the north, a liaison role has been created. “We have created the role of ambassadors, people we recruit from different departments, so that this becomes an integrated approach, with at least one designated point of contact within each service” (I-15, Head of the Ecological Transition Unit). Others have incorporated the communication department into the committee to promote the collaboration of patients and users through the dissemination of sustainability-related information.

The organizational structure related to environmental management in smaller centres was not clearly identified by participants working in clinical care. However, they perceive that the commitment to reducing environmental impact is primarily driven by higher levels of management and is progressing towards a more environmentally committed healthcare system. “I believe that centres, their managers and their departments are increasingly concerned about environmental issues, showing a growing commitment and implementing measures to support the transformation of infrastructures and processes” (I-1, Occupational Health Nurse).

### 3.3. Environmental Analysis and Financing

The monitoring and identification of aspects for improvement aimed at implementing strategies to reduce environmental impact include, among other measures, the annual calculation of the carbon footprint (which participants reported as mandatory), as well as the preparation of reports including data on resource consumption, emissions, audit results and environmental management objectives to be achieved, alongside other key indicators.

Six participants agreed that higher hospital energy consumption is largely attributable to air-conditioning, especially in clinical units that require specific regulator conditions to operate, with operating theatres and Intensive Care Units (ICUs) cited as key examples. These units require precise parameters of temperature, humidity, filtration and pressure to ensure safe patient care, resulting in higher energy consumption. Due to the demand for care, energy consumption cannot be reduced in these areas; however, participants indicated that efficiency could be increased by renewing and improving the performance of air-conditioning equipment. “The age of the building makes it difficult to implement energy efficiency measures” (I-14, Environmental Management Officer). “Without upgrading facilities or equipment, it is not possible to achieve improvements” (I-2, Deputy Economic Director).

Carrying out this type of situational analysis, together with identifying improvement measures to be implemented by healthcare institutions, is essential for accessing financial support, which participants considered a key factor in promoting more environmentally responsible healthcare. “I am not sure whether there is a specific budget for this purpose, but it is important that funding is allocated to implement measures so that the healthcare environment is as healthy as possible” (I-1, Occupational Health Nurse).

According to the participants, the Spanish public administration is organized into budgetary chapters in which public expenditure and revenue are classified according to their nature. In chapters related to investments, funding from the European Regional Development Fund (ERDF) aimed at reducing the carbon footprint was highlighted, particularly for the co-financing of infrastructure and improvements in energy efficiency. In this way, healthcare institutions that have sought such funding have been able to apply by presenting a prior cost–benefit analysis, which includes data on consumption, emissions, payback period, useful life and maintenance. “I believe that photovoltaic solar panels will meet all of the organization’s expectations, including for those health centres considering their installation” (I-12, Deputy Director of Services and Centres Management, Andalusian Health Service).

However, the proposals submitted by institutions must pass numerous administrative filters in order to be approved and access these funds, which often results in significant delays in the implementation of measures. “One of the main problems within public administration is the slow processing of applications” (I-12, Deputy Director of Services and Centres Management, Andalusian Health Service).

In addition to the ERDF funds, the participants mentioned the Next Generation EU (NGEU) funds, a European instrument more strongly focused on ecological transition and digitalisation. These funds are time-limited, offering a defined timeframe for the implementation of sustainable strategies; therefore, institutions benefiting from NGEU funding tend to prioritize the most urgent needs.

### 3.4. Interinstitutional Collaboration for Environmental Management

To ensure coherence between institutional policies and operational actions, it is necessary to consider the involvement of other entities in environmental and health management.

Green public procurement could drive the transition toward a more sustainable healthcare system by incorporating environmental criteria into public procurement processes and including carbon footprint requirements in tender specifications [36]. However, its transformative potential is often underestimated, as priority is given to the acquisition of goods and services that are more economically advantageous “When a contract is awarded, environmental clauses are considered, but cost is given much greater priority if it is economically more advantageous” (I-8, Environmental Management Officer). This situation indicates that there is no mandatory requirement within the healthcare sector to procure goods and services that comply exclusively with environmental criteria.

On the other hand, collaboration between healthcare institutions and other local entities highlights an ideal partnership for developing sustainable strategies. Regarding access to healthcare facilities through less polluting modes of transport, the data reveal differences according to the size of the institution. In smaller healthcare facilities, coordination exists between the city’s public transport provider and staff working schedules, enabling more efficient and sustainable commuting.

However, findings from larger healthcare institutions suggest a lack of support from the local administration. Although these institutions promote the use of sustainable transport, they require corresponding support from local authorities through the development of infrastructure and environmental improvements to effectively implement the strategic plans designed by the healthcare centre. As two participants explained: “There are plans to create a bicycle lane access, but this is beyond our control. Many people use this service, but the existing road conditions make it unsafe, even walking to the hospital can be dangerous” (I-11, Deputy Director of Industrial Processes and Services); “We are one of the largest hospitals in Catalonia, but we do not have direct access to high-capacity public transport [such as the metro], which puts us at a disadvantage” (I-20, Environmental Management Officer).

With regard to the management of waste generated by healthcare practices, differentiated strategies were observed depending on the type of institution. Small centres or units, such as ORPCs, include clauses in their procurement processes to ensure efficient waste management, reflecting an explicit institutional commitment to good practices. In contrast, the hospitals analyzed (*n* = 14) rely primarily on external providers for the collection and disposal of hazardous waste, while waste comparable to municipal solid waste is managed in collaboration with local municipal services. However, challenges arise that affect the implementation of these practices. The location of some facilities limit access for collection vehicles, and there are no clear solutions to facilitate waste separation. As one participant noted: “At the Huércal-Overa healthcare centre, there are no waste containers outside; the cleaner who disposes of cardboard has to walk 500 metres to the nearest container” (I-10, environmental management technician). Another participant highlighted the frustration and disconnection this situation generates: “Some municipalities have not placed paper, cardboard or plastic containers near health centres. So why should I segregate paper and plastic if there is nowhere to dispose of them?” (I-18, Environmental Management Officer). These experiences indicate that the lack of adequate infrastructure and logistical support can lead to resistance and demotivation among staff, limiting the effectiveness of implemented practices.

Nevertheless, there are regions of the country where intersectoral collaboration is well established, such as in northern Spain, where collaboration with the business sector enables the management of organic waste through its conversion into animal feed, and in eastern Spain, where collaboration exists with an NGO that removes used cooking oil for resale, with the proceeds allocated to the training of people with Down syndrome.

## 4. Discussion

The disparity in implementation the legal framework is significant, characterized by progress in waste management, but limitations in internal governance, financing, and sustainable procurement.

The heterogeneity in the implementation of a multilevel regulatory framework within the Spanish healthcare sector may limit the full transition of current healthcare institutions toward more sustainable models, as previously described by Vallée [37]. However, the outcome of regulatory implementation does not depend solely on the national political organization; it is also influenced by global geopolitical dynamics.

In this context, the announcement of the withdrawal of the United States (US) from the Paris Agreement, a key international treaty aimed at addressing climate change, and its support for the use of fossil fuels [38,39] have contributed to a global weakening of climate commitments, potentially slowing the decarbonisation of sectors such as healthcare [40]. Similarly, the war between Russia and Ukraine has led to an increase in the price of gas and electricity in Europe [41], which may force the reallocation of healthcare system budgets, with an impact on investments in sustainable infrastructure and energy efficiency programmes.

Conversely, the adoption of certifications such as ISO or EMAS was identified as a factor strengthening the quality of environmental management, in line with evidence associating these instruments with improvements in performance and institutional reputation [42,43]. Nevertheless, the results show that their implementation is not widespread due to the economic and organizational demands associated with their maintenance, consistent with findings from other studies [43,44]. This finding suggests that, although these certifications constitute effective tools for advancing toward more environmentally responsible healthcare systems, their implementation largely depends on the availability of structural resources and sustained managerial commitment.

Regarding EMS, previous studies have documented improvements in the environmental performance of healthcare institutions of up to 28% following their implementation [45]. Although the present study highlights the partial existence of EMS with varying levels of maturity, it did not assess the extent of improvement resulting from their implementation, thereby limiting the ability to evaluate their transformative impact in quantitative terms. However, advanced waste management practices were observed, which may be attributable to the presence of specific regulations in this area [46]. This finding reinforces the notion that the establishment of regulatory frameworks promotes institutional action. Nevertheless, challenges persisted in logistical coordination with the authorities responsible for final disposal, underscoring that sustainable strategies require a full supply chain perspective rather than solely the implementation of internal protocols, as noted in the existing literature [47].

With regard to internal governance, the presence of environmental managers and environmental management committees fosters cross-sectoral collaboration and enhances awareness among staff, in line with European evidence highlighting the importance of formal structures to integrate sustainability into hospital management [45]. However, the absence of environmental departments led by or including healthcare professionals suggests a limited transmission of sustainability commitments from senior management to frontline care levels or insufficient training, as noted by Luque-Alcaraz et al. [48] in their study. In this context and considering that healthcare professionals constitute a key group in driving climate action [48], providing sustainability training to these professionals could represent a strategic approach to reducing the environmental impact of the healthcare system.

On the other hand, the higher male representation in environmental management positions (64.7%) compared to women (35.3%) may be attributable to sociocultural factors that hinder women’s access to leadership roles [49] which contrasts with studies linking female leadership to a greater adoption of innovative and sustainable strategies [50,51]. Although our study did not directly assess the impact of gender on environmental performance, this finding suggests the value of incorporating a gender perspective into environmental governance policies within the healthcare sector.

With regard to obtaining funding, the limited staffing levels within environmental management departments and competition between institutions make it difficult to access this aid, which can lead to an uneven implementation of sustainability strategies [52], despite funding being a key factor in reducing the environmental impact of healthcare institutions. This situation also reveals a critical paradox: there are European funds (ERDF, Next Generation EU) aimed at decarbonisation, but administrative bureaucracy acts as a significant barrier. Managers perceive that delays in the processing of applications put at risk the implementation of urgent improvements in energy efficiency, which are vital for high-consumption areas such as operating theatres and ICUs.

At the same time, the establishment of strategies that allow cost savings in daily operations should not compromise patient safety. There are studies that have investigated this issue, showing that improving energy efficiency while maintaining patient safety is possible [53,54].

Regarding interinstitutional collaboration, the results of the study by López-Rodríguez et al. align with the findings of the present study concerning the relationship between healthcare institutions and other public administrations [55]. Participant testimonies highlight that healthcare professionals recognize their responsibility to carry out certain environmentally responsible practices, such as waste segregation. However, the lack of coordination between healthcare institutions and other entities may pose a barrier to effective environmental performance. This finding reinforces the theory that actions carried out by professionals alone are insufficient; rather, a mature organizational culture is required to provide sustainable healthcare that protects human health, consistent with other studies [56]. Additionally, coordination between healthcare services and the business sector is identified as a key factor for more sustainable healthcare through nurse-led initiatives, such as the recycling of bottles in the neonatology ward, which are transformed into gardening materials and outdoor furniture in collaboration with a private company, the recycling of compression bandages, or the redistribution of medical equipment to low-income countries [57,58].

Regarding the purchase of medical devices, the scarcity of companies and suppliers that offer environmentally sustainable resources could be decisive in limiting the acquisition of products, goods and services that are fully environmentally responsible. In procurement processes, the results obtained indicate that environmental criteria are taken into consideration; however, economic considerations continue to prevail, consistent with the findings of Smith-Rodríguez et al. [59]. The application of tools such as the Analytic Hierarchy Process (AHP) has made it possible to compare medical devices in the purchasing process of hospitals in Buenos Aires, facilitating the acquisition of more environmentally responsible alternatives [59], an approach that could also be applied within Spanish healthcare institutions.

### Study Strengths and Limitations

This study has certain limitations that must be taken into account. First, a purposive sampling combined with snowball sampling was used, which could leave out participants from other levels of care or from other centres in the same geographical area. Furthermore, this type of sampling allowed participants to be recruited through institutional channels, which may have influenced them to present their institution’s strategies in an overly positive light. Although it was hypothesized that management dynamics could be shared by institutions in the same Autonomous Community, the results showed that the operational practices may vary between centres. In addition, the decentralization of healthcare in Spain may limit the generalizability of certain findings across the national context.

Secondly, although effective meetings was used as a starting point to learn about the general functioning of environmental management in the healthcare sector and relevant regulatory frameworks, it may not have been possible to access internal reports or strategic plans, which warrants further investigation. Furthermore, the results are specific to the Spanish context, meaning they may not be readily transferable to different settings or populations. Therefore, it would be valuable to conduct comparative studies with other healthcare systems in Southern Europe or with countries following the Beveridge model, in order to identify similarities, differences, and contextual factors that may influence the implementation of sustainability strategies in healthcare.

On the other hand, our results highlight the commitments of healthcare institutions to mitigating their environmental impact for the protection of global health outcomes, contributing to objectives such as the United Nations 2030 Agenda, the European Green Deal and the sustainability strategies of the Spanish National Healthcare System. Future research could focus on the implementation of care protocols, professional training and feasible organizational changes within public healthcare institutions, and analyze environmental management not only through technical indicators, but also in terms of its real impact on patients, professionals and communities.

## 5. Conclusions

Spanish National Health System (NHS) institutions demonstrate a clear commitment to environmental sustainability through the adoption of frameworks, regulations, and voluntary environmental management certifications, in line with applicable international, national, and regional legislation. However, the study reveals that the implementation of environmentally responsible strategies is heterogeneous, influenced both by the structural characteristics of the healthcare system and by the organizational and resource diversity of each institution.

The implementation of Environmental Management Systems (EMS) enables institutions to monitor their environmental impact and improve performance, although maturity levels and the professional profiles responsible vary considerably. The existence of comprehensive environmental management plans at the regional level helps guide institutions toward more sustainable practices, yet the results show that their effectiveness depends on factors such as the availability of specialized personnel, the flow of information from management to frontline professionals, and adequate financial resources.

Critical barriers limiting the effective implementation of sustainable measures were also identified: bureaucratic procedures in accessing funding, limited intersectoral collaboration, insufficient integration of environmental criteria in public procurement, and unequal participation of professionals in environmental management roles.

This study provides evidence on how Spanish healthcare institutions navigate the tension between their moral obligation to protect health and the environmental impact of their activities within a decentralized healthcare system. The results indicate that providing sustainable healthcare requires not only regulations and certifications but also organizational support, human and financial resources, and an institutional culture that incorporates ethical considerations into daily management.

Based on these findings, policymakers are recommended to increase staffing and training in environmental management units, including sustainability education for clinical professionals; optimize administrative processes for accessing environmental funding, reducing delays that hinder the implementation of urgent measures; strengthen intersectoral coordination, incorporate environmental criteria into public procurement, and foster partnerships with local and private entities; and integrate sustainability into clinical career progression through certifications that recognize environmentally responsible practices. In addition, future research should further explore the differentiated impacts of environmental pollution on vulnerable populations within the Spanish healthcare context. In particular, incorporating a gender perspective would allow for a more nuanced understanding of exposure patterns, health outcomes, and access to mitigation and adaptation measures

These actions could help Spanish hospitals move toward a healthcare model that is more sustainable, ethically coherent, and environmentally responsible.

## Figures and Tables

**Table 1:** Script for semi-structured interviews.

To what extent is your school committed to environmental sustainability? Which areas or people show the most commitment? How? Are there pro-environmental leaders, commissions or specific groups within the institution? How do they work and how do they work? What regulations or guidelines on sustainability are currently applied? (i.e., European regulations, ISO standards, Green Hospital construction standards, regional and state laws, indicators for certifications, protocols, programmes, intervention or research projects, etc.). Are there any sources of funding? Are there mobility plans? Are there practices for managing and/or reducing healthcare waste?

**Table 2:** Delphi Panel Expert Features.

Code	Age	Sex	Professional Profile and Occupation *	Workplace	Experience
Expert 1	48	Male	Nurse Nursing supervisor *	Virgen Macarena Hospital	22 years
Expert 2	43	Female	Economist and professor Environmental Economics *	University of Sevilla	10 years
Expert 3	42	Female	Sustainability Coordinator Assistant manager of education, training and sustainability action *	University of Mexico	23 years
Expert 4	47	Male	Nurse	Hospital Virgen Macarena	23 years
Expert 5	43	Male	Chemical Engineer Department of Chemical and Environmental Engineering *	University of Sevilla	20 years

* Experts’ occupation.

**Table 3:** Characteristics of the participants.

Interviewee	Sex/Age (Years)	Position Held	Workplace	Experience
I-1	Male/33	Occupational Health Nurse	ORPC of Jaén (Jaén)	10 years
I-2	Female/48	Deputy Economic Director (Meso-Management)	Hospital Universitario Virgen de las Nieves (Granada)	22 years
I-3	Female/49	Pain Therapy Nurse	Centro Clínico Biotronic Salud (Granada)	10 years
I-4	Female/48	Head of the Occupational Risk Prevention Service	Hospital Universitario Torrecárdenas (Almería)	25 years
I-5	Female/33	Occupational Health Nurse	ORPC of Huelva (Huelva)	10 years
I-6	Male/50	Deputy Director of Economic, Administrative and General Services (Meso-Management)	Hospital Universitario Puerta del Mar (Cádiz)	22 years
I-7	Male/32	Environmental management technician (Meso-Management)	Hospital Universitario Virgen Macarena (Sevilla)	4 years
I-8	Female/68	Environmental Management Officer (Meso-Management)	Hospital Universitario Reina Sofía (Córdoba)	20 years
I-9	Female/50	Environmental management technician (Meso-Management)	Hospital General de Riotinto (Huelva)	5 years
I-10	Female/54	Environmental management technician (Meso-Management)	Hospital La Inmaculada (Huércal-Overa, Almería)	1 year
I-11	Male/54	Deputy Director of Industrial Processes and Services (Meso-Management)	Hospital Universitario de Poniente (El Ejido, Almería)	21 years
I-12	Male/59	Deputy Director of Services and Centers Management of the Servicio Andaluz de Salud (Macro-Management)	Servicios centrales del Servicio Andaluz de Salud (Sevilla)	>10 years
I-13	Male/42	Environmental Management Officer (Meso-Management)	Hospital Alto Guadalquivir (Jaén)	20 years
I-14	Male/46	Environmental Management Officer (Meso-Management)	Hospital Universitario Príncipe de Asturias (Madrid)	4 years
I-15	Female/53	Head of the Ecological Transition Unit (Meso-Management)	Hospital Universitario de Navarra (Pamplona)	1 year
I-16	Male/47	Environmental Management Officer (Meso-Management)	Hospital Universitario Virgen de la Arrixaca (Murcia)	17 years
I-17	Male/46	Deputy Director of Sustainability Management and Ecological Transition (Macro-Management)	Servicio Canario de Salud (Islas Canarias)	3 years
I-18	Male/62	Environmental Management Officer (Meso-Management)	Hospital General de Valencia (Valencia)	43 years
I-19	Female/25	Environmental management technician (Meso-Management)	Hospital Germans Trias i Pujol (Barcelona)	2 years
I-20	Male/46	Environmental Management Officer (Meso-Management)	Hospital Germans Trias i Pujol (Barcelona)	7 years
I-21	Male/60	Head of Management Control, Data Exploitation and Registration Service (Meso-Management)	Hospital General de Riotinto (Huelva)	5 years

**Table 4:** Differences and similarities of external regulations applied.

	ISO 14001 ^a^	ISO 50001 ^a^	EMAS ^b^
Scope	International	International	UE ^c^
Character	Voluntary	Voluntary	Voluntary
Goal	Reduce environmental impact	Improve energy performance	Promote environmental excellence and public transparency
Scope of application	Organizations of different natures ^1^	Organizations of different natures	Organizations of different natures
Certification period	Every 3 years	Every 3 years	Every 3 years
Environmental statement	Optional	Optional	Annual, mandatory, public and externally verified
Audits period	Internal: at the institution’s discretion External: annual	Internal: at the institution’s discretion External: annual	Internal: at the institution’s discretion, within a period not exceeding 3 years. External: every 3 years.

^1^ Industries, small and medium-sized enterprises, public administration or other entities. ^a^ International Organization for Standardization. The ISO 14001 refers to environmental management and ISO 50001 to energy management; ^b^ Eco-Management and Audit Scheme; ^c^ Union Europe.

## Data Availability

The data that support the findings of this study are available upon request from the corresponding authors.

## Supplementary Materials

The following supporting information can be downloaded at: https://www.mdpi.com/article/10.3390/healthcare14060753/s1, Table S1: Consolidated Criteria for Reporting Qualitative Studies (COREQ): 32-Item Checklist.

## Abbreviations

The following abbreviations are used in this manuscript:

WHO	World Health Organization
GHG	GreenHouse Gas
EU	Europe Union
SUSTAinsHealth	Development of an ethical and gender-sensitive model for the implementation of sustainability measures in healthcare centres and institutions
AACC	Autonomous Communities
NHS	National Health System
ORPC	Occupational Risk Prevention Center
ISO	International Organization for Standarization
EMS	Environmental Management System
EnMS	Energy Management System
EMAS	Eco-Management Audit Scheme
PESMA	Plan Estratégico de Salud y Medioambiente
ACSA	Agencia de Calidad Sanitaria de Andalucía
ERDF	European Regional Development Fund
NGEU	Next Generation EU
