# Supplementary material for: Decarbonizing the Spanish Health System: A Qualitative Study on the Implementation of Environmental Regulations and Management Strategies in Health Institutions
Source: Healthcare (Basel). 2026 Mar 17;14(6):753. doi: 10.3390/healthcare14060753 (PMC13026714; doi:10.3390/healthcare14060753)
Supplement: Supplementary file 1 [file healthcare-14-00753-s001.zip › Table S1.pdf]

Table S1. Consolidated criteria for reporting qualitative studies (COREQ): 32-item checklist

No	Item	Guide questions/description	Response
		Domain 1: Research team and reflexivity	
		Personal Characteristics	
1.	Interviewer/facilitator	Which author/s conducted the interview or focus group?	The interviews were conducted by the three authors (LMP, BB, RDC).
2.	Credentials	What were the researcher's credentials? E.g. PhD, MD	BB and RDC were Phd. LMP was Phd Student.
3.	Occupation	What was their occupation at the time of the study?	BB and RDC were researchers and LMP was nurse.
4.	Gender	Was the researcher male or female?	All researches were female.
5.	Experience and training	What experience or training did the researcher have?	BB and RDC had experience in carrying out qualitative research. LMP has been trained to conduct interviews and data analysis.
		Relationship with participants	
6.	Relationship established	Was a relationship established prior to study commencement?	No, there wasn't.
7.	Participant knowledge of the interviewer	What did the participants know about the researcher? e.g. personal goals, reasons for doing the research	Name, occupation, reasons for doing the research.
8.	Interviewer characteristics	What characteristics were reported about the interviewer/facilitator? e.g. Bias, assumptions, reasons and interests in the research topic	Name, occupation, contact method, reasons for doing the research.
		Domain 2: Study design	
		Theoretical framework	
9.	Methodological orientation and Theory	What methodological orientation was stated to underpin the study? e.g. grounded theory, discourse analysis, ethnography, phenomenology, content analysis	Phenomenological approach with a discourse and content analysis.
		Participant selection	
10.	Sampling	How were participants selected? e.g. purposive, convenience, consecutive, snowball	Purposive and snowball sampling.
11.	Method of approach	How were participants approached? e.g. face-to-face, telephone, mail, email	Telephone and email
12.	Sample size	How many participants were in the study?	21
13.	Non-participation	How many people refused to participate or dropped out? Reasons?	None
		Setting	
14.	Setting of data collection	Where was the data collected? e.g. home, clinic, workplace	In workplace and by video-calling.

15.	Presence of non- participants	Was anyone else present besides the participants and researchers?	In some cases, there was another person working near
16.	Description of sample	What are the important characteristics of the sample? e.g. demographic data, date	4 healthcare professionals and 17 environmental managers (15 meso-level management, 2 macro-level management).
		Data collection	
17.	Interview guide	Were questions, prompts, guides provided by the authors? Was it pilot tested?	Yes, they were. / Yes, it was.
18.	Repeat interviews	Were repeat inter views carried out? If yes, how many?	No, they weren't.
19.	Audio/visual recording	Did the research use audio or visual recording to collect the data?	Audio recording.
20.	Field notes	Were field notes made during and/or after the interview or focus group?	Yes, they were (field notes).
21.	Duration	What was the duration of the interviews or focus group?	Average 90 minutes
22.	Data saturation	Was data saturation discussed?	Yes, it was.
23.	Transcripts returned	Were transcripts returned to participants for comment and/or correction?	No, it wasn't.
		Doman 3: Analysis and findings	
		Data analysis	
24.	Number of data coders	How many data coders coded the data?	One (LMP).
25.	Description of the coding tree	Did authors provide a description of the coding tree?	Yes, we did.
26.	Derivation of themes	Were themes identified in advance or derived from the data?	Themes were derived using both methods.
27.	Software	What software, if applicable, was used to manage the data?	None.
28.	Participant checking	Did participants provide feedback on the findings?	No, we didn't.
		Reporting	
29.	Quotations presented	Were participant quotations presented to illustrate the themes/findings? Was each quotation identified? e.g. participant number	Yes, there were. / Yes, there was.
30.	Data and findings consistent	Was there consistency between the data presented and the findings?	Yes, there was.
31.	Clarity of major themes	Were major themes clearly presented in the findings?	Yes, they were.
32.	Clarity of minor themes	Is there a description of diverse cases or discussion of minor themes?	Yes, there is.

Tong, A.; Sainsbury, P.C. Consolidated Criteria for Qualitative Research Reporting (COREQ): A 32-Item Checklist for Interviews and Focus Groups. *Int J Qual Health Care.* **2007**, *19*, pp. 349-57, 2007.
